# Voltammetric detection of Neuropeptide Y using a modified sawhorse waveform

**DOI:** 10.1007/s00216-024-05373-y

**Published:** 2024-06-25

**Authors:** Nadiah Alyamni, Jandro L. Abot, Alexander G. Zestos

**Affiliations:** 1 Department of Biomedical Engineering, The Catholic University of America, Washington, D.C. 20064 USA; 2 Department of Mechanical Engineering, The Catholic University of America, Washington, D.C. 20064 USA; 3 Department of Chemistry, American University, Washington, D.C. 20016 USA

**Keywords:** Fast-scan cyclic voltammetry (FSCV), Neuropeptide Y (NPY), Carbon fiber microelectrodes (CFMEs), Modified sawhorse waveform (MSW), Adsorption, Biofouling, Co-detection

## Abstract

The hormone Neuropeptide Y (NPY) plays critical roles in feeding, satiety, obesity, and weight control. However, its complex peptide structure has hindered the development of fast and biocompatible detection methods. Previous studies utilizing electrochemical techniques with carbon fiber microelectrodes (CFMEs) have targeted the oxidation of amino acid residues like tyrosine to measure peptides. Here, we employ the modified sawhorse waveform (MSW) to enable voltammetric identification of NPY through tyrosine oxidation. Use of MSW improves NPY detection sensitivity and selectivity by reducing interference from catecholamines like dopamine, serotonin, and others compared to the traditional triangle waveform. The technique utilizes a holding potential of −0.2 V and a switching potential of 1.2 V that effectively etches and renews the CFME surface to simultaneously detect NPY and other monoamines with a sensitivity of 5.8 ± 0.94 nA/µM (*n* = 5). Furthermore, we observed adsorption-controlled, subsecond NPY measurements with CFMEs and MSW. The effective identification of exogenously applied NPY in biological fluids demonstrates the feasibility of this methodology for in vivo and ex vivo studies. These results highlight the potential of MSW voltammetry to enable fast, biocompatible NPY quantification to further elucidate its physiological roles.

**Graphical Abstract:**

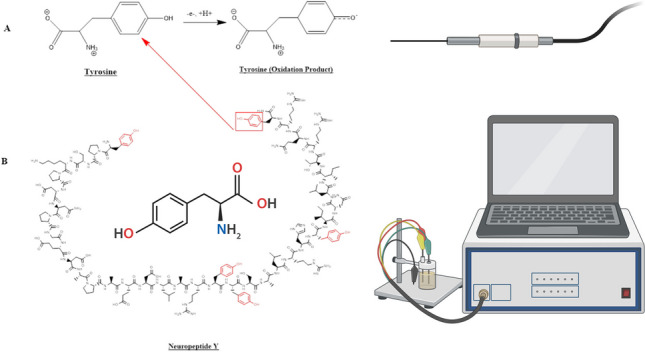

## Introduction

Numerous human diseases and disorders can be attributed to an inherent perturbation in the equilibrium of biomolecules and neurotransmitters (NT). However, distinguishing and detecting these molecules remains a challenging task [1]. Neuropeptides, which are composed of multiple amino acid residues, are an alternative class of neurohormones that play a crucial role in the transmission and modulation of neurological impulses. It is essential to achieve high temporal and spatial resolution for the accurate and biocompatible measurement of neuropeptides [1]. Neuropeptides are commonly released with other neurotransmitters, thereby presenting a significant obstacle in their selective and sensitive measurement [2, 3]. NPY is composed of 36 amino acids and is one of the foremost significant human neurotransmitters [2]. It plays a role in the regulation of physiological processes, including the manifestation of depressive symptoms [4], hunger/satiety, feeding [5], memory [6], fear [7], and stress [4, 8].

Several methods have been used to measure and quantify NPY. Previous studies have employed mass spectrometry in combination with chromatographic separation to quantify NPY. Studies have utilized high-performance liquid chromatography-mass spectrometry (HPLC-MS) for the analysis of NPY in plasma samples [9]. However, these methodologies have exhibited certain constraints in terms of their sensitivity, hence posing a challenge in acquiring diagnostic data, particularly when dealing with small sample volumes [3]. Another work has reported a limit of detection of 1 nM for NPY in plasma samples, with an analysis time of approximately 25 min per sample, and this method focused solely on quantifying NPY [10]. Other studies have utilized mass spectrometric detection, which enabled high sensitivity and selectivity, but often had relatively lower spatiotemporal resolution than other methods [11]. Immunoassays exhibit a notable capacity to attain high levels of sensitivity, as evidenced by their ability to detect concentrations as low as picomolar levels. NPY has also been isolated from brain and sweat samples via microdialysis [3]. However, this required off-line analysis of the collected dialysate, involving either HPLC with electrochemical detection or immunoassays [3]. Moreover, Vocat et al. reported detecting NPY levels as low as 0.25 pM in sweat patch samples. These immunoassays measured total immunoreactive NPY for the full immunoassay, which includes NPY metabolites and fragments that cross-react with the antibody [13].

The use of electrochemical detection techniques has garnered considerable attention as a prompt and effective approach for quantifying biomolecules. The utility of capillary electrophoresis with electrochemical detection (CE-EC) allows for rapid assessment of neurochemicals. Analyzing NPY is challenging due to sensitivity issues, as reported by Crespi et al. [12]. However, the tyrosine residues within NPY peptides have higher oxidation potentials (~1.0 V) than do commonly measured compounds such as catecholamines (~0.6 V), potentially enabling their co-detection alongside monoamines through the discrimination of their oxidation peaks [15]. Thus, establishing precise techniques for these less common, yet biologically significant, peptides is essential and very useful. The electrochemical detection of neuropeptides containing tyrosine, such as enkephalins (ENKs), has added challenges that has recently been addressed. It is hypothesized that these molecules are found at relatively low concentrations in the extracellular space for short durations, and they adsorb to the electrode surface where oxidation occurs. Several electrochemical methods such as the modified sawhorse waveform have been used to measure oxytocin in animal models such as zebrafish using fast-scan cyclic voltammetry (FSCV) [13, 14]. Endogenous peptides such as met-enkephalin and leu-enkephalin have previously been measured electrochemically through the oxidation of tyrosine residues [15–17]. While the MSW has been extensively characterized for smaller molecules and neuropeptides, its application to the detection of larger neuropeptides, such as Neuropeptide Y (NPY), represents a novel endeavor. NPY has been measured with several electrochemical techniques such as square wave voltammetry and electrochemical impedance spectroscopy (EIS) [1, 18]. Moreover, electrodes have been modified with aptamers [19], polypyrrole [20], and methylene blue dye to enhance the sensitivity and selectivity of the measurement of NPY with the aforementioned techniques [1, 18]. Therefore, the measurement of the tyrosine-containing NPY with electrochemical techniques seems promising for the sensitive and selective measurement of this peptide.

Here, we develop an assay for the electrochemical measurement of NPY with CFMEs and FSCV. We utilized MSW for the direct and minimally invasive detection of NPY, achieving high spatiotemporal resolution. The MSW waveform encompasses two separate scan rates during each anodic sweep, while also introducing a short period of holding at the switching potential (1.2 V). We address two key challenges: minimizing electrode fouling difficulties and boosting the chemical resolution in the detection of peptides that contain tyrosine. Through this work, we have expanded the range of possible applications for carbon fiber microelectrodes to measure several tyrosine-containing peptides with FSCV. As a result, these electrodes can now be utilized for the detection of not just small compounds but also larger and more intricate molecules, such as neuropeptides, including NPY [21]. MSW was used to measure NPY through targeted oxidation of its tyrosine residue. In this work, we demonstrated that NPY measurement was enhanced utilizing the MSW waveform as opposed to the traditional waveform. We also showed that NPY was adsorption-controlled to the surface of CFMEs and had stable detection for several hours in addition to being fouling resistant. We co-detected NPY in complex solutions with other monoamines and catecholamines such as dopamine, serotonin, norepinephrine, and others. Moreover, NPY was also measured in complex biological fluids illustrating proof-of-concept potential measurement in animal models. As opposed to conventional techniques, we have developed a fast, sensitive, selective, biocompatible, and minimally invasive assay with high spatiotemporal resolution to measure NPY. This will potentially enhance the measurement of NPY and further understanding of its physiological role in vivo.

## Materials and methods

### Chemicals

The experimental components utilized in the study were comprised of high-purity reagents. Tyrosine and dopamine, both with a purity level surpassing 98.0%, were obtained from Sigma-Aldrich (St. Louis, MO). NPY was obtained from GenScript Biotech (Piscataway, NJ). The electrochemical studies were performed using a phosphate-buffered saline (PBS) solution, which consisted of specific concentrations of several components. These components included 131.5 mM NaCl, 3.25 mM KCl, 1.2 mM CaCl_2_, 12.5 mM NaH_2_PO_4_, 1.2 mM MgC1_2_, and 2.0 mM Na_2_SO_4_. The pH of the solution was adjusted to 7.4.

### CFME fabrication

The fabrication process of carbon fiber microelectrodes (CFMEs) occurred by using T-650 carbon fibers (Goodfellow, UK), which are derived from polyacrylonitrile (PAN). A T-650 carbon fiber strand with a diameter of approximately 7 μm was carefully aspirated into a glass capillary (A-M Systems, Sequim, WA) with an inner diameter of 0.68 mm and an exterior diameter of 1.2 mm. A vacuum pump (Gast, Model DOA-P704-AA, Benton Harbor, MI) was utilized to aspirate the fiber into a glass capillary. Following that, the glass was tapered using a micropipette vertical capillary puller (Narishige, PC-100, Tokyo, Japan). The carbon fiber’s protruding end was cut to a length of approximately 100 to 150 μm from the tapered side of the glass capillary. The glass-carbon fiber interface was sealed with EPON 828 epoxy resin and diethylenetriamine (DETA) hardener. The epoxy was cured for 4 h at a temperature of 125 °C. A 4 M KCl solution was used to backfill the electrodes to create an electrical connection with the electrode holder.

### Fast-scan cyclic voltammetry (FSCV)

A WaveNeuro fast-scan cyclic voltammetry (FSCV) potentiostat was equipped with a 5 MΩ headstage and supplied by Pine Instruments (Durham, NC). High-definition cyclic voltammetry (HDCV) software, in conjunction with the PC1e-6363 multifunction I/O device produced by NI (Austin, TX), was used to collect and analyze the data. A conventional triangle waveform was utilized, where the holding potential was set at −0.4 V and the switching potential was set at 1.3 V. A scan rate of 400 V/s was employed, with a silver-silver chloride reference electrode (Ag/AgCl) maintained at a potential of 0.197 V and a frequency of 10 Hz. The MSW was also introduced at a frequency of 10 Hz. The initial resting potential of the waveform was set at −0.2 V and subsequently increased to +0.7 V at a rate of 100 V/s. It proceeded to increase to a value of +1.2 V while being subjected to a higher scan rate of 400 V/s. The potential was sustained for a brief period of 3 ms, followed by a rapid decrease to −0.2 V, with a scan rate of 100 V/s.

To maintain a consistent and uninterrupted flow of solution, the buffer solution was administered to the electrode tip at a steady rate of 1 mL/min, facilitated by the NE-300 Just Infusion Syringe Pump (New Era Systems, Farmingdale, NY) and a FSCV flow injection analysis flow cell (Pine Research, Durham, NC). In each experimental iteration, 0.2-mL aliquots of NPY were injected into the system. The electrode was allowed to equilibrate with the waveform applied for a minimum duration of 30 min, which was allotted for each electrode prior to conducting in vitro measurements within the flow cell.

### Statistical analysis

GraphPad Prism 9 was utilized for the preparation of figures and execution of linear regression computations, encompassing the calculation of slope and *R*^2^ values. This was important for obtaining the statistical significance between the urine samples and the buffer. Several data sets were normalized by dividing all of the peak oxidative currents by the largest current in the data set to normalize to “1” or “100%.” The normalized current was obtained by dividing each peak oxidative current by the highest current to account for differences in protruding length of the carbon fiber from electrode to electrode.

## Results and discussion

### Neuropeptide Y measurement

**Fig. 1:**
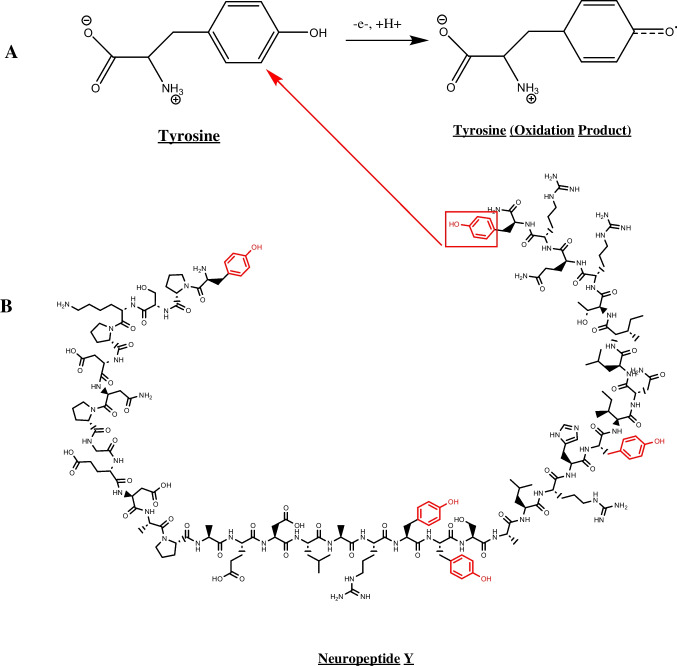
Oxidation of tyrosine in the structure of NPY. **A** The redox mechanism showing tyrosine being oxidized to a resonance stabilized free radical intermediate. **B** The full 36 amino acid sequence of NPY, with the five tyrosine residues highlighted in red. Electrochemical detection of NPY relies on the oxidation of these tyrosine residues

NPY is an endogenous peptide consisting of 36 amino acids arranged linearly, with tyrosine groups located at both ends of the molecule [22]. The tyrosine residues within the NPY structure serve as crucial redox moieties with significant functional importance. The electrochemical oxidation-reduction of tyrosine is characterized by a proton-coupled electron transfer (PCET) mechanism. The PCET process arises due to the distinct acid dissociation constants observed for tyrosine in its oxidized state (pKOX < 0) and reduced state (pKRED ∼ 10). This phenomenon is significant within the context of the limited pH range in which proteins can maintain their structural stability and catalytic activity [15, 23]. Tyrosine is oxidized into a free radical intermediate through a concerted mechanism (Fig. 1).

### MSW measurements of NPY

We employed the MSW with various voltages, scan rates, and hold times. The MSW waveform was designed to maintain a holding potential of −0.2 V during the scan. The scan was initiated at a rate of 100 V/s and continued until it reached a transition potential of 0.7 V. After reaching 0.7 V, there is a transition in the scan rate, which increases to 400 V/s. This elevated scan rate is maintained until reaching the switching potential of 1.2 V where it is held constant for a specific duration of 3 ms. Subsequently, the scanning process continues and scans down until reaching a voltage of −0.2 V, with a constant rate of 100 V/s. As shown in previous studies [13], holding the potential at 1.2 V for 3 ms improved adsorption and promoted effective electron transfer kinetics on the surface of carbon fiber microelectrodes (CFMEs). Maintaining a potential of 1.2 V facilitates the oxidation of tyrosine and enhances the flow of electrons between the analyte and the surface of the CFME. At this potential, the waveform can etch the electrode and renew the surface, which enhances sensitivity and prevents biofouling [13, 17]. This is supported by the distinct shape of the cyclic voltammogram (CV) peak. Several studies have shown NPY to be present and physiologically active at nanomolar and micromolar levels, which makes this assay relevant for the measurement of NPY [24–27].

**Fig. 2:**
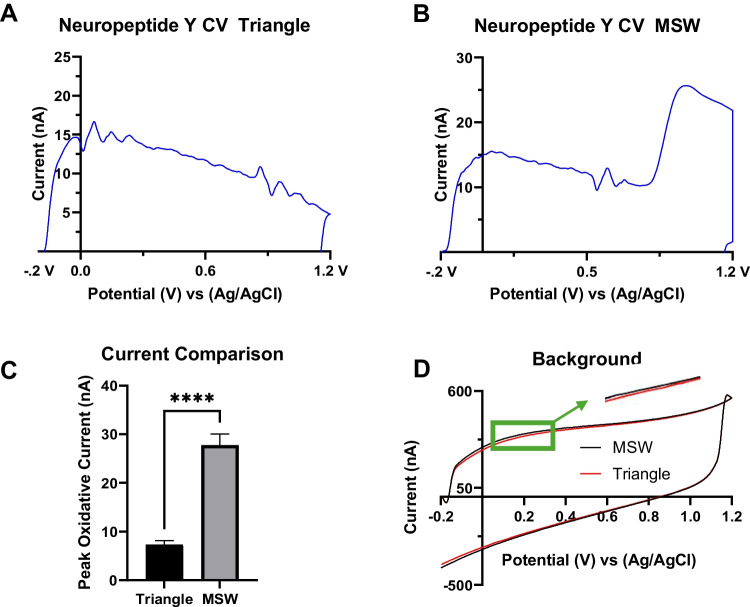
Electrochemical characterization of NPY. **A** Cyclic voltammogram of NPY (20 µM) using a triangle waveform. **B** Cyclic voltammogram of NPY (20 µM) measured using MSW. **C** Statistical analysis comparing the peak oxidative currents obtained using the triangle and MSW waveforms, respectively, that show significantly higher peak oxidative current for the MSW waveform (*n* = 6, *p* < 0.0001). **D** Background charging current for comparison between CFMEs applied with the MSW and triangle waveforms. The non-faradaic background charging capacitive current magnitudes are comparable to one another.

We observe an oxidation peak at about 1.0 V and a secondary peak at about 0.4 V, which could be the result of an acid shift due to several acidic amino acid moieties included in NPY. This secondary peak could be utilized to distinguish NPY from the electrochemical detection of other tyrosine-containing peptides such as oxytocin [13], leu-enkephalin [17], and met-enkephalin [16]. The triangle waveform was not able to detect and measure NPY, and produced higher noise and lower peak oxidative currents at a concentration of 20 μM (Fig. 2A). We observe higher peak oxidative currents at around 1.1 V for an electrode measuring NPY at the same concentration of 20 μM when applied with the MSW waveform (Fig. 2B). Therefore, we have shown that applying the CFME with MSW, as opposed to the conventional triangle waveform, has significantly enhanced NPY measurements with FSCV (Fig. 2B). When compared across multiple electrodes, CFMEs applied with the MSW yield significantly higher peak oxidative currents and better peak shape for tyrosine detection than those applied with the traditional triangle waveform (Fig. 2C, n = 6, *p* < 0.0001). The background charging capacitive currents of electrodes applied with the MSW and triangle waveforms were of comparable magnitude (Fig. 2D). Therefore, we expect that the electrodes had comparable surface roughness and area when applied with the two waveforms. Any differences in peak oxidative current and CV shape was hypothesized to arise from faradaic current arising from direct electrron transfer at the surface of the electrode and not from any changes in the background charging current, which are similar in magnitude.

The selectivity of an analytical technique is a crucial factor that determines its applicability and reliability, particularly in complex biological matrices. The detection of NPY relies on the oxidation of its tyrosine residues, which are also present in various other peptides and electroactive biomolecules. These peptides could potentially contribute to the electrochemical signal, leading to inaccurate measurements of NPY. However, the presence of other redox active amino acid residues within the peptide, such as tyrosine, phenylalanine, tryptophan, cysteine, and potentially others, offers the possibility of selectively measuring peptides with this technique. The unique properties of NPY that enhance its selectivity and distinguish it from other peptides are primarily related to its size and the presence of multiple tyrosine residues. Research has demonstrated that the larger size of NPY contributes to its adsorption-controlled oxidation kinetics [16]. NPY contains five tyrosine residues, which are electroactive and contribute to the oxidation signal. The presence of multiple tyrosine residues in NPY can result in a unique electrochemical signature compared to peptides with fewer or no tyrosine residues [28]. As opposed to oxytocin, NPY has a secondary oxidation peak at approximately ~0.4 V and a primary oxidation peak at ~1.0 V; therefore, we are able to differentiate NPY from other tyrosine-containing peptides.

### Concentration experiments

**Fig. 3:**
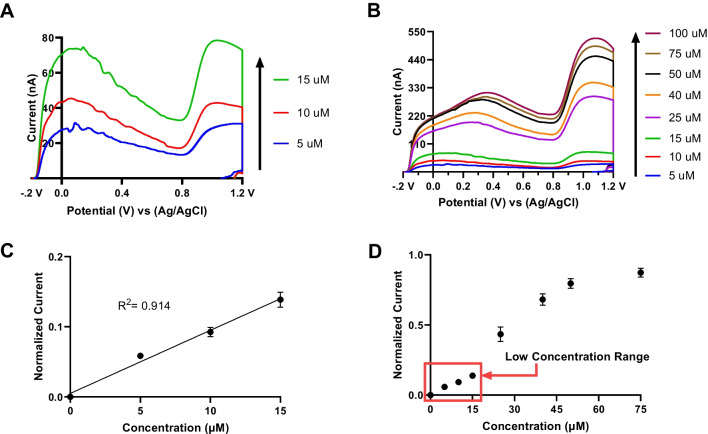
Electrochemical characterization of NPY at varying concentrations. **A** Cyclic voltammograms of NPY at lower concentrations ranging from 5 to 15 µM. **B** Cyclic voltammograms of NPY at higher concentrations ranging from 5 to 100 µM. **C** Normalized current versus NPY concentration and linear fit from 5 to 15 µM (*R*^2^ = 0.914) whereby the current is normalized to 1 by dividing by the largest current in the data set. **D** Normalized current versus all NPY concentrations tested (*n* = 6), including the higher concentration range where there is a deviation from linearity

We then attempted to vary the concentration to measure adsorption control dynamics at the surface of NPY at the surface of CFMEs. In typical cyclic voltammograms (CVs) of NPY oxidation utilizing the MSW waveform at different concentrations with current increasing with concentration, we observed an increasing peak oxidative current of the CVs upon increasing the concentration of NPY from 5 to 15 µM (Fig. 3A). An increasing peak oxidative current of the CVs upon increasing the concentration was observed (Fig. 3B). Moreover, a linear response is observed between 5 and 15 µM (Fig. 3C) (*R*^2^ = 0.914). This illustrates that peak oxidative current is linear with respect to concentration up to 15 µM as denoted by the Randles-Sevcik equation for voltammetric analysis. At concentrations higher than 15 µM (Fig. 3D), the plot deviates from linearity forming a discernible asymptotic curve. As such, we hypothesize that NPY adsorption sites become saturated and NPY becomes more diffusion-controlled at the electrode surface, which can explain the deviation from linearity and asymptotic curve at higher concentrations. At higher concentrations, NPY and other molecules become saturated at the surface of the electrode and, hence, fill up adsorption sites at the electrode surface, which promote more diffusion control at higher concentrations [28].

### Scan rate and stability experiments

**Fig. 4:**
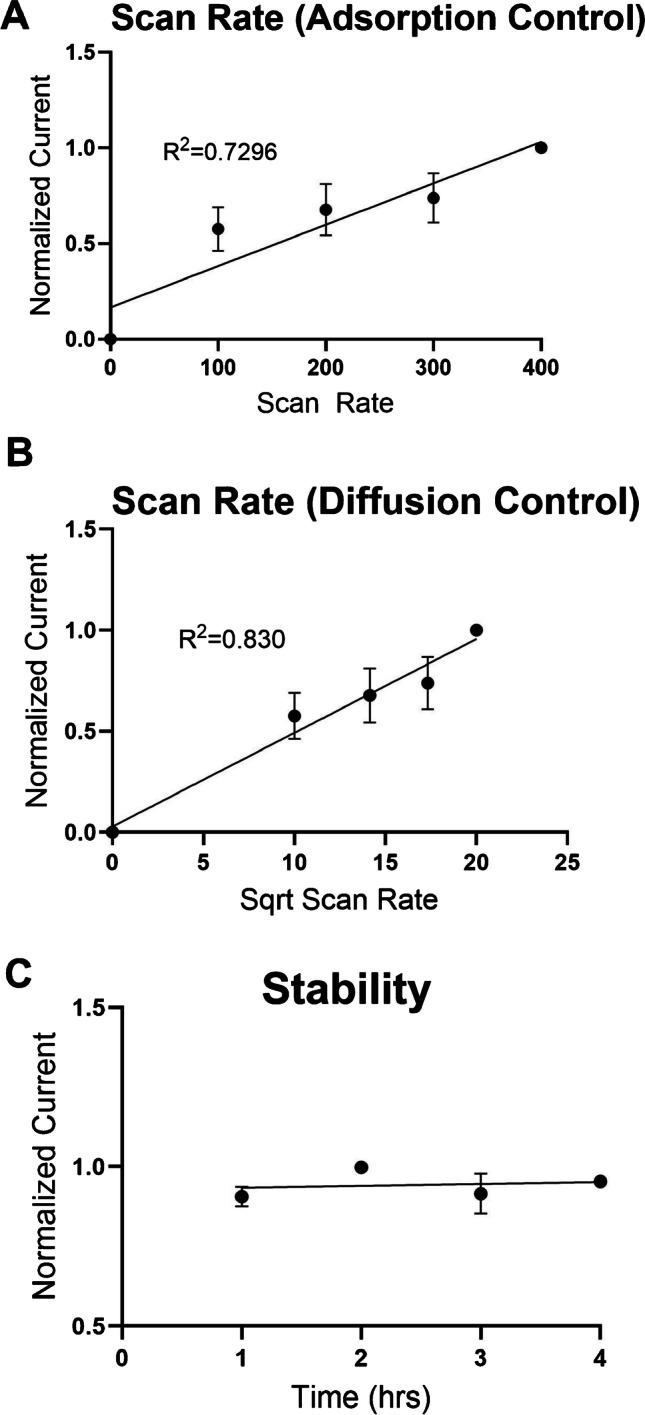
**A** Normalized peak oxidative current vs. scan rate. The scan rate is proportional and linear with respect to the peak oxidative current between 100 and 400 V/s (*R*^2^ = 0.729). **B** Normalized peak oxidative current vs. square root of scan rate. **C** The measurement of NPY remains stable during a 4-h time period. Every hour, NPY concentrations (20 μM) are injected into the flow cell and measured with CFMEs and FSCV (*n* = 4)

We then varied the scan rate (upward scan) of the first upward scan from 100 to 400 V/s in 100 V/s increments. Upon plotting peak oxidative current vs. scan rate (Fig. 4A), we observed a linear relationship between peak oxidative current and scan rate (*R*^2^ = 0.729). Therefore, we hypothesize that the kinetics of NPY are adsorption-controlled at the electrode surface. Adsorption-controlled processes allowed for increased sensitivity in comparison to diffusion-controlled kinetics where peak oxidative current would be proportional to the square root of scan rate. When the molecule adsorbs to the microelectrode surface, it enhances electron transfer and sensitivity, which can produce lower limits of detection [29, 30]. Moreover, we also observed strong linear correlation between peak oxidative current and the square root of scan rate (R² = 0.83) (Fig. 4B). Therefore, we hypothesize that there is partial adsorption and diffusion control of NPY at the surface of the electrode.

Using the MSW, we observed a stable peak oxidative current response over 4 h, while successfully detecting a concentration of 5 μM of NPY (Fig. 4C). Here, the electrode was continuously applied with the MSW and was stable over 4 h. It is worth noting that the 4-h duration corresponds to the standard timeframe typically employed for conducting in vivo measurement. Applying the MSW etches and renews the surface of the carbon fiber, which could potentially increase background capacitance and drift [31, 32]. Therefore, the electrode is allowed to equilibrate for approximately 30 min to prevent any additional electrode drift throughout the duration of the experiment. The mechanism of adsorption is postulated to be an electrostatic attraction of opposite charges where positively charged amino acid residues of NPY are electrostatically attracted to and adsorb on the negatively charged electrode surface. Moreover, the phenyl group of tyrosine could also undergo π-π stacking with the surface of the graphitic carbon fiber microelectrode as well [33]. Maintaining a stable response over 4 h is important as it is the typical duration of ex vivo and in vivo studies [34]. The application of the MSW enables the detection of NPY, even at lower concentrations, and establishes the capability for the possible detection of endogenous NPY in living organisms at physiological levels. Moreover, the stability experiment (Fig. 4C) provides additional evidence supporting the consistent accuracy of NPY detection when utilizing the MSW. These findings demonstrate that there were no significant variations observed over a 4-h duration.

### Fouling experiments

Fouling occurs when an analyte polymerizes and forms non-conductive coatings on the electrode surface, preventing sites for subsequent adsorption and, hence, lowers the sensitivity of electrochemical detection [35–38]. Molecules such as serotonin and one of its major downstream metabolites, 5-hydroxyindoleacetic acid (5-HIAA), produce extremely reactive radicals during the oxidation process. These radicals then polymerize to form non-conductive coatings on the surface of the carbon fiber microelectrode (CFME), which can diminish analyte detection [39]. These coatings obstruct electron transfer, causing electrode fouling and reducing peak oxidative current.

**Fig. 5:**
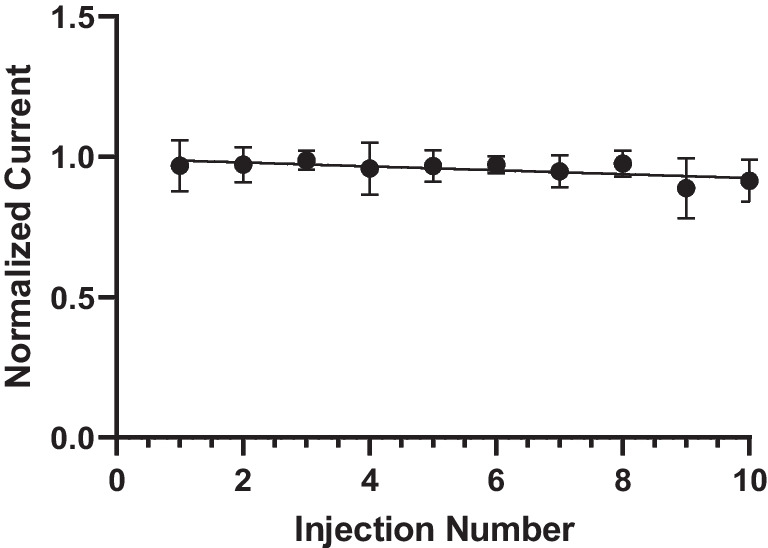
Using the modified sawhorse (MSW) waveform, 20 μM of NPY was repeatedly injected into the flow cell and measured with CFMEs. After 10 injections, there was no discernible decrease in peak oxidative current in comparison to the first injection when plotting injection number vs. normalized current, which was obtained by dividing each peak oxidative current by the highest current to account for differences in protruding length of the carbon fiber from electrode to electrode. No statistically significant differences were observed between first and last injection (ns, *t*-test, *p* = 0.332, *n* = 5)

We attempted to determine whether other molecules such as NPY also foul the electrode surface. Following ten consecutive injections of 20 μM NPY onto the CFME in the flow cell (Fig. 5) shows that when using the MSW, there is no detectable decrease in peak oxidative current over repeated ten repeated injections of NPY (approximately 5 min). The application of the MSW effectively reduces fouling at the electrode surface. It is hypothesized that holding the upper potential limit at 1.2 V for 3 ms prevents biofouling by etching the electrode and renewing the electrode surface, which prevents the coating of the electrode surface in non-conductive polymer films. Previous studies have revealed that applying this waveform may etch and renew the microelectrode surface [32, 40–43], which would make it an appropriate waveform for NPY detection and the prevention of fouling at the electrode surface as well.

Prior research has shown that scanning to higher potentials than 1.2 V favors the renewal of the electrode surface through etching [13]. This etching process breaks carbon-carbon bonds, increases the surface roughness, which causes the electroactive surface area and aspect (surface to volume) ratio to grow, and functionalizes the electrode surface with negatively charged oxide groups such as oxides, hydroxy, carbonyl, carboxyl, ketones, and others, which can adsorb positively charged cations such as monoamines [32]. Analyte fouling at the electrode surface is efficiently prevented by regenerating the electrode surface through electrochemical etching. This continuous renewal of the electrode surface prevents non-conductive polymers from coating the electrode surface, ensuring that the electrode stays functional and prevents coating with non-conductive polymer throughout the duration of the experiment [44].

We also performed a fouling experiment where we applied the electrode with the MSW in vitro. In the flow cell, we injected 20 μM of NPY every 30 s for a total of ten injections for a total run time of 300 s (5 min). We observed no statistically significant difference in peak oxidative current vs. injection number when plotting the normalized peak oxidative current vs. injection number or time (Fig. 5) (*p* = 0.33). Therefore, we hypothesize that NPY does not foul the surface of the electrode when applied with the MSW.

### Co-detection experiments

**Fig. 6:**
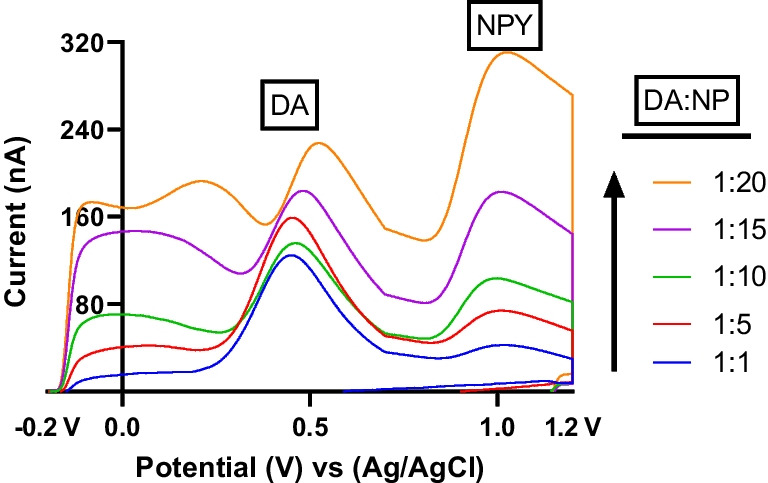
Dopamine (DA) and NPY were co-detected in a complex mixture utilizing the MSW waveform at concentrations ranging from 1 μM DA and 1-20 μM NPY (1:1–1:20)

We then employed the MSW to investigate the electrochemical responses of dopamine (DA) and NPY in complex mixtures throughout a concentration range of 1 μM DA to 20 μM of NPY. The results consistently showed unique and defined oxidation potentials, with NPY oxidizing at approximately +1.1 V and dopamine oxidizing at +0.5 V. As we increased the concentration of NPY, the peak oxidative current of the NPY CV increased, while that of dopamine remained relatively constant, which further illustrated the co-detection of both DA and NPY as they oxidized at substantially different potentials (Fig. 6). Therefore, it was possible to co-detect and differentiate both molecules from one another in a complex mixture.

When the concentrations of DA and NPY were equal, we were not able to observe the CV current peak for NPY due to a relatively higher sensitivity for DA at the electrode surface. Peak oxidative currents for NPY increased significantly when NPY was present at higher concentrations relative to DA (at a 1:20 ratio). When NPY concentrations were at the lower end, noise levels rose as the concentration levels approached the limit of detection. This phenomenon can mostly be attributed to the relatively facile nature of dopamine oxidation at CFMEs, which is more amenable to electron transfer at the electrode surface due to its smaller size and faster electron transfer kinetics where the catechol moiety is oxidized to a quinone and then reduced back to a catechol. Furthermore, when co-detecting dopamine with NPY or tyrosine, our experiments revealed a notable separation of oxidation peaks. The variable rates of heterogeneous electron transfer of dopamine and NPY when interacting with carbon fiber microelectrodes (CFMEs) could explain this phenomenon. One potential explanation for the separation of the dopamine and NPY peaks is the use of multiple scan rates during the MSW application. Within the theoretical window, favorable to dopamine oxidation, the scan rate is maintained at 100 V/s, while it increases to 400 V/s for NPY. Because dopamine has a faster electron transfer rate, its oxidation peak exhibits more significant alterations in response to changes in scan rate. As the scan rate is reduced, the dopamine oxidation peak shifts to more negative potentials. In contrast, when the scan rate is constant throughout, the peak oxidative position for tyrosine or NPY remains rather stable. This work is important as the carbon fiber microelectrode sensors could potentially be used to measure NPY in complex heterogeneous environments in the brain that also contain other biologically relevant molecules such as monoamine neurotransmitters. Therefore, it is important to be able to co-detect and differentiate NPY from other monoamine neurotransmitters such as dopamine.

### Co-detection of NPY with other monoamine neurotransmitters

**Fig. 7:**
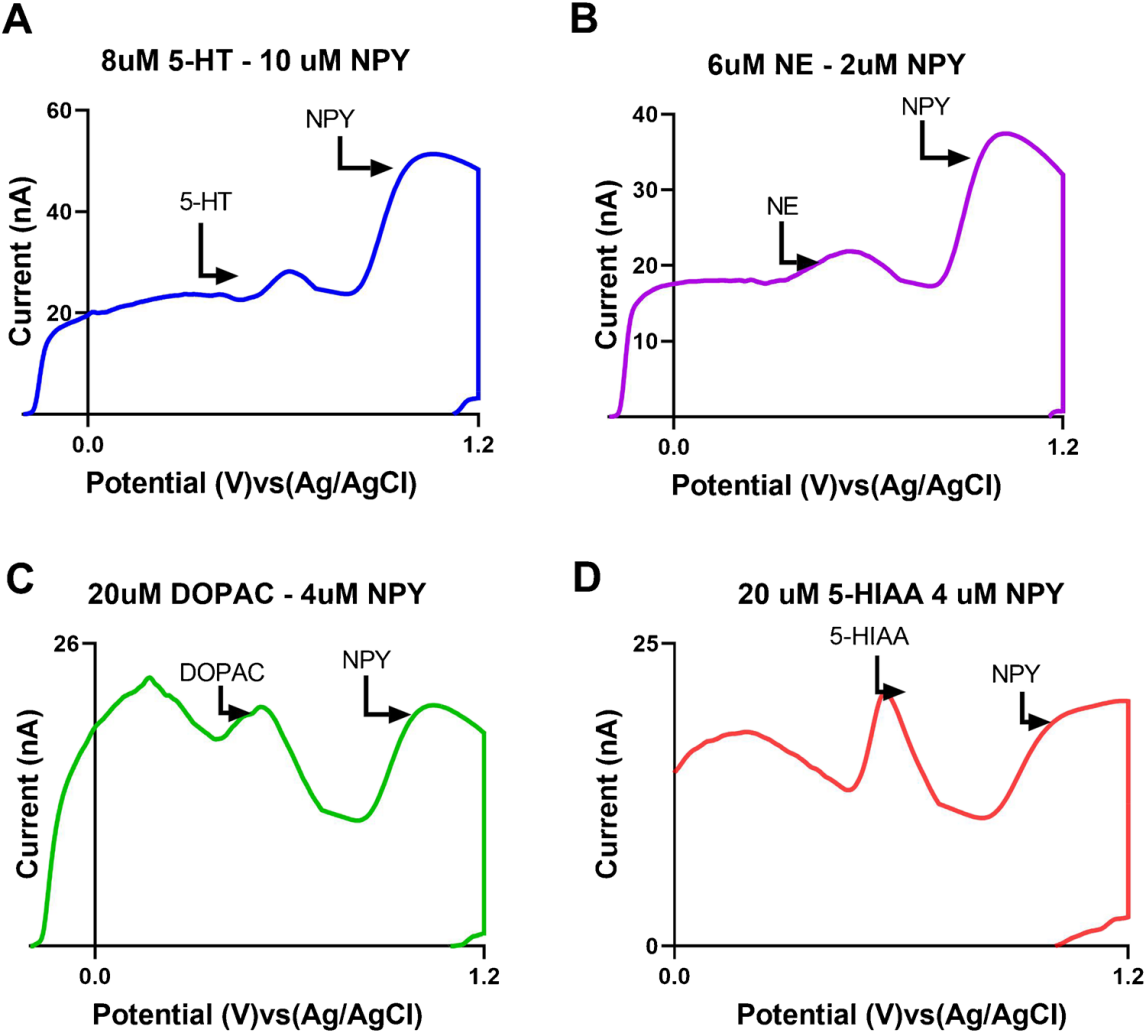
Selectivity characterization of NPY detection with carbon fiber microelectrodes (CFMEs) using fast-scan cyclic voltammetry (FSCV) in the presence of potentially interfering small molecule analytes. **A** Serotonin (5-HT) interference tested at ratios of 4:5 relative to 10 μM NPY. **B** Norepinephrine (NE) interference tested at a ratio of 3:1 relative to 2 μM NPY. **C** 3,4-Dihydroxyphenylacetic acid (DOPAC) interference tested at a ratio of 5:1 relative to 4 μM NPY. **D** 5-Hydroxyindoleacetic acid (5-HIAA) interference tested at a ratio of 5:1 relative to 4 μM NPY. Higher analyte ratios were used for the anionic species DOPAC and 5-HIAA compared to the cationic neurotransmitters. The data indicate that NPY, being a larger peptide, can only be detected at higher concentrations relative to the tested small molecule interferents. NPY is often found at similar concentrations and brain regions with other brain monoamines; therefore, the co-detection of these molecules is important for understanding brain anatomy and function [46–48]

In addition to the dopamine co-detection experiments, we performed interference studies with a variety of other neurochemicals including 8 μM serotonin (5-HT) and 10 μM NPY (Fig. 7A), 6 μM norepinephrine (NE) and 2 μM NPY (Fig. 7B), 20 μM 3,4-dihydroxyphenylacetic acid (DOPAC) and 4 μM NPY (Fig. 7C), and 20 μM 5-hydroxyindoleacetic acid (5-HIAA) and 4 μM NPY(Fig. 7D). Using MSW, we were able to co-detect NPY in complex mixtures with several other monoamine amine neurotransmitters due to the peak separation of the NPY CV oxidation peak from that of the other monoamine interferents. As with dopamine, the other monoamine neurotransmitters oxidize (and have peak oxidation potentials) at approximately 0.6 V, while the peak oxidative potential is approximately 1.1 V for NPY. DOPAC and 5-HIAA were tested at higher concentrations because they are anionic (negatively charged) and, thus, have lower sensitivity than positively charged cations (dopamine and serotonin) because they are electrostatically repelled from the negatively charged (holding potential) and oxide-rich carbon electrode surface.

In comparison to 5-HIAA and DOPAC, NPY consistently showed the greater oxidation peaks among the other chemicals studied. This observation can be explained by the fact that both DOPAC and 5-HIAA are carboxylic acids and anions at a physiological pH. 5-HIAA has been known to generate extremely reactive radicals during the oxidation process [45] and has been shown to foul the electrode surface [40]. These radicals are most likely responsible for the fouling of 5-HIAA at the electrode surface. The monoamine neurotransmitters display comparable electrochemical CV behavior to dopamine as they are similar in size and structure to dopamine and, hence, have comparable redox properties as well. Higher concentrations (20 μM) of DOPAC and 5-HIAA (the carboxylic acid–functionalized analogues of dopamine and serotonin, respectively) were utilized because they are anionic, negatively charged, and do not adsorb to the surface of the electrode because they are electrostatically repelled from the negatively charged electrode surface and are more diffusion controlled. Therefore, higher concentrations must be tested to compensate for this in order to yield comparable peak currents to NPY. The monoamine cations (dopamine, serotonin, and norepinephrine) are cationic, positively charged, and adsorb to the electrode surface, which are more sensitive and produce higher peak currents. This allows for the measurement of relatively lower concentrations as opposed to other molecules that do not adsorb to the electrode surface.

### Measurement in biological fluid

**Fig. 8:**
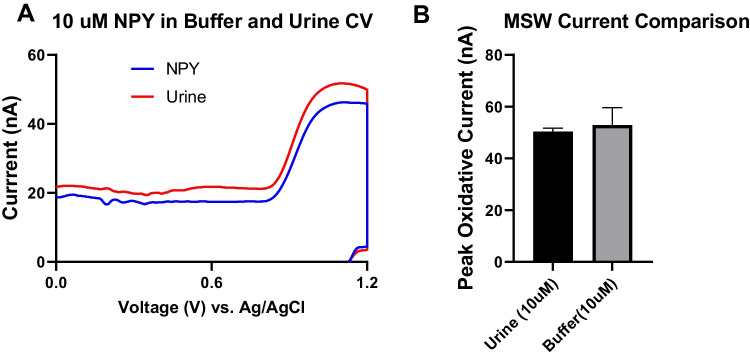
**A** Measurement of NPY in urine. **B** Comparison between buffer and urine CVs. There was no decrease in NPY detection upon testing in urine vs. the buffer (*t*-test, *p* = 0.74, ns, *n* = 5)

Lastly, we performed a proof-of-concept experiment to detect exogenously applied NPY in a biological urine sample. We performed FSCV testing for NPY diluted in buffer and NPY spiked into a urine sample, both at a concentration of 10 μM. The results showed that NPY could be detected even in the presence of urine (Fig. 8). The cyclic voltammograms (CVs) clearly showed a strong, identifiable peak that matched the characteristics observed with in vitro testing. Comparing the same electrodes, there was no statistically significant decrease in peak oxidative current of the CVs between measurements of NPY in buffer or urine as shown in the example CVs (Fig. 8A) and the data across multiple electrodes (Fig. 8B). Redox active interferents such as uric acid found in urine did not hinder NPY detection by CFMEs in urine as opposed to in buffer. The measurement of NPY in urine is an established assay as it serves as a biomarker for urinary tract disorders and renal disease, which makes this biological fluid a suitable model for the measurement of NPY in real samples [49–51]. This illustrates the robustness of our electrochemical assay with CFMEs when immersed in biological fluids such as urine. Measuring NPY levels in urine could potentially have biological relevance, as NPY has been reported to be excreted in urine and proposed as a biomarker for certain conditions like obesity, metabolic disorders, and renal diseases [52, 53].

## Conclusions

In summary, we have shown that the MSW applied onto CFMEs with FSCV was utilized to successfully measure NPY via the oxidation of tyrosine. The use of the MSW proved to be a critical assay in enhancing NPY detection sensitivity due to the use of a holding potential of 1.2 V, which permitted a more efficient electron transfer processes and prevented biofouling. Notably, our findings reveal that holding the potential at 1.2 V renewed the electrode surface faster than the conventional triangle waveform and allowed more time for analyte oxidation. This significant electrochemical etching of the surface led to a significant improvement in the sensitivity of NPY detection and allowed for the successful co-detection of NPY with multiple monoamines. Furthermore, applying the MSW onto CFMEs increased NPY selectivity by reducing interference from catecholamines such as dopamine and serotonin, thus allowing for co-detection and the distinct separation of NPY’s oxidation peak from those of the monoamine neurotransmitters. The enhanced detection of NPY in urine with this assay indicates the practical utility of this technique for potential real-time in vivo monitoring or in real biological samples.

## Declarations

### Source of biological material

Carolina Biological Supply
